# Age-Related Reductions in Tactile and Motor Inhibitory Function Start Early but Are Independent

**DOI:** 10.3389/fnagi.2019.00193

**Published:** 2019-07-30

**Authors:** Marit F. L. Ruitenberg, Kaitlin E. Cassady, Patricia A. Reuter-Lorenz, Mark Tommerdahl, Rachael D. Seidler

**Affiliations:** ^1^Department of Experimental Psychology, Ghent University, Ghent, Belgium; ^2^Department of Health, Medical and Neuropsychology, Leiden University, Leiden, Netherlands; ^3^Department of Psychology, University of Michigan, Ann Arbor, MI, United States; ^4^Department of Biomedical Engineering, University of North Carolina, Chapel Hill, NC, United States; ^5^Department of Applied Physiology and Kinesiology, University of Florida, Gainesville, FL, United States

**Keywords:** aging, cortical inhibition, motor control, tactile processing, somatosentory function, lifespan

## Abstract

Aging is associated with declines in motor and somatosensory function. Some of these motor declines have been linked to age-related reductions in inhibitory function. Here we examined whether tactile surround inhibition also changes with age and whether these changes are associated with those in the motor domain. We tested a group of 56 participants spanning a wide age range (18–76 years old), allowing us to examine when age differences emerge across the lifespan. Participants performed tactile and motor tasks that have previously been linked to inter- and intra-hemispheric inhibition in the somatosensory and motor systems. The results showed that aging is associated with reductions in inhibitory function in both the tactile and motor systems starting around 40 years of age; however, age effects in the two systems were not correlated. The independent effects of age on tactile and motor inhibitory function suggest that distinct mechanisms may underlie age-related reductions in inhibition in the somatosensory and motor systems.

## Introduction

Maintaining functional independence is a major concern in aging. Somatosensory and motor function have been linked to central nervous system changes with age including brain volumetric declines, altered patterns of brain functional activity and connectivity, and reductions in neurotransmission (for reviews see Seidler et al., 2010; Levin et al., 2014; Sala-Llonch et al., 2015; Heft and Robinson, 2017; Maes et al., 2017). Within the somatosensory tactile system, Von Békésy (1967) provided early evidence for cortical lateral inhibition of surrounding receptive fields upon skin contact. Such surround inhibition results in a sharper neural representation of stimuli and has also been shown to extend to contralateral homologous representations (Halliday and Mingay, 1961). Animal work has demonstrated that such inhibition is a γ-aminobutyric acid (GABA)-dependent phenomenon, as administration of a GABA receptor antagonist expands receptive fields in the somatosensory cortex (Hicks and Dykes, 1983; Tremere et al., 2001). It is currently unclear how healthy aging affects this phenomenon, and whether potential age-related changes start to occur around the same time as those reported for changes in GABA concentration (i.e., beginning in the third decade of life; Gao et al., 2013) and for changes in bimanual coordination (i.e., beginning in the fifth decade; Boisgontier et al., 2018). It has been well established that two-point tactile discrimination distance increases with age (Vieira et al., 2016), but this test assesses both peripheral and central sensory function.

Here, we determine tactile thresholds for a given target finger while either a neighboring or the contralateral digit are simultaneously stimulated; this allows us to test tactile surround inhibition in individuals across a wide range of ages. In young adults, stimulation on a neighboring finger has been shown to increase tactile thresholds for the target finger (i.e., intrahemispheric inhibition; Nguyen et al., 2013), as the former stimulation causes the receptive field of the target finger to be inhibited. For older participants, we hypothesize that low amplitude stimulation on a neighboring finger will, somewhat paradoxically, decrease tactile thresholds due to “spillover” effects. Such “spillover” effects have been demonstrated in the motor domain as well, where inhibition of a specific effector results in inhibition of neighboring motor representations (Greenhouse et al., 2015; Duque et al., 2017). For our tactile task, we expect that the low amplitude stimulation will pre-activate the neural area representing the target finger in older adults, such that detection of the target stimulus is facilitated. In contrast, higher amplitude stimulation on a neighboring finger should lead to increased inhibition and consequently interfere with detection (see Nguyen et al., 2013), especially for older adults. We expect to observe the same pattern for stimulation on a contralateral finger (i.e., interhemispheric inhibition).

Inter- and intrahemispheric inhibition in the motor system change with age as well (for a review see Levin et al., 2014). For example, using transcranial magnetic stimulation (TMS) we have shown increased interhemispheric facilitation and a trend for reduced interhemispheric inhibition in older compared to young adults (Fling and Seidler, 2012). These measures were differentially correlated with the ability to perform asynchronous bimanual actions in young and older adults (Fling and Seidler, 2012). Similar findings have been reported in a TMS study by Fujiyama et al. (2012), who observed that inhibitory function was reduced in older adults and that this reduction was linked to age differences in performance on an interlimb coordination task. Additionally, older adults exhibit more expansive motor cortical representations (Carp et al., 2011; Bernard and Seidler, 2012) and reduced intracortical inhibition (Peinemann et al., 2001). Thus, in the current study, we also administered a motor tapping task requiring movement of one digit, or movement of two digits asynchronously allowing us to evaluate functional inter- and intrahemispheric inhibition in the motor system. Previous work has shown that bimanual asynchronous tapping requires interhemispheric inhibition by callosal connections between motor cortices, such that movement of one hand is associated with activation in contralateral primary motor cortex (M1) and inhibition in ipsilateral M1 (Fling et al., 2011; Duque et al., 2017). We hypothesized that performance of asynchronous movements of two digits on the same hand (i.e., unimanual action; intrahemispheric inhibition) or of two homologous digits (i.e., bimanual action, see Fling et al., 2011; interhemispheric inhibition) would be negatively associated with age and positively correlated with tactile surround inhibition measures. This would indicate parallel age differences in somatosensory and motor inhibitory function; sampling across a wide age range will illustrate the lifespan trajectory of declines.

## Materials and Methods

### Participants

Fifty-six healthy volunteers (23 males, 33 females) ranging in age from 18 to 76 years were enrolled in the study. They all performed the tactile tasks and 37 of these participants additionally performed the motor tapping task (16 male, age range 19–76 years). Only a subset of participants performed the motor task because it was added after data collection had started, and not all participants met standard eligibility criteria for MRI scanning (as explained below, the motor task was performed in the scanner). All participants self-reported being right-handed and having normal or corrected-to-normal hearing and vision. None had a history of psychiatric or neurological disorders. Written informed consent was obtained from all participants, who received monetary compensation for their participation. The study was approved by the Institutional Review Board of the University of Michigan.

### Apparatus

For the tactile tasks, we used two portable vibrotactile stimulators (CM5, Cortical Metrics, LLC) to deliver stimulation to the participants’ fingers (Holden et al., 2012). Vibrations were delivered *via* 5 mm diameter probes that stimulated the index and middle fingers of each hand. Stimulus presentation, timing, and response registration were controlled by the Brain Gauge software application (Cortical Metrics, LLC). Participants responded *via* a tiny computer mouse (“TinyMouseT Optical”; Chester Creek Technologies, Inc., Duluth, MN, USA) placed under their right thumb. For the motor tapping task, we used two Fiber Optic Response Claws (one for each hand; Psychology Software Tools) to record participants’ finger-tapping responses. The presentation and timing of finger-specific stimuli and recording of the responses were controlled by Cogent 2000 software^1^.

### Experimental Design and Procedure

We administered the Montreal Cognitive Assessment (MoCA; Nasreddine et al., 2005), the Digit Symbol Substitution Test (DSST), and the Purdue Pegboard test (right hand, left hand, bimanual, and assembly tasks, three runs per task) to evaluate participants’ general cognitive and motor abilities.

#### Tactile Tasks

Participants performed a series of computerized tasks that assessed their tactile processing capacity. We used the following six metrics: (1) simple reaction time (sRT); (2) choice reaction time (cRT); (3) static detection threshold (DT); (4) dynamic DT; (5) dynamic DT with concurrent stimulation to another finger on the same hand (intrahemispheric condition); and (6) dynamic DT with concurrent stimulation to a finger on the other hand (interhemispheric condition).

The RT and DT tasks have been used and described in detail in previous studies assessing tactile processing in both healthy and clinical populations (e.g., Zhang et al., 2011; Puts et al., 2013, 2014; Nguyen et al., 2014; Francisco et al., 2015). In the RT tasks, participants received a vibration (duration 40 ms, amplitude 200 μm, frequency 25 Hz) to one finger and were instructed to click a mouse button as soon as they felt it. In the sRT task any click was sufficient (10 trials per finger of each hand), while in the cRT task participants additionally had to indicate on which finger they felt the vibration by clicking either the left or right mouse button (five trials per finger of each hand). RT was recorded for each trial. In the cRT task, response accuracy was additionally recorded. In the static threshold task, participants received a vibration to either the index or middle finger of one hand (10 trials per finger) and were asked to indicate the finger on which the vibration was delivered. The stimulus amplitude started at 15 μm on the first trial and was adjusted on subsequent trials using an adaptive staircase algorithm depending on the accuracy of the participant’s response. In the dynamic threshold task, participants were instructed to select the finger on which the vibration was delivered as quickly as possible. The stimulus started at an amplitude of 0 μm and increased at a rate of 2 μm/s (seven trials per hand). The stimulus amplitude at the time of the participant’s response was recorded for each trial.

**Figure 1:**
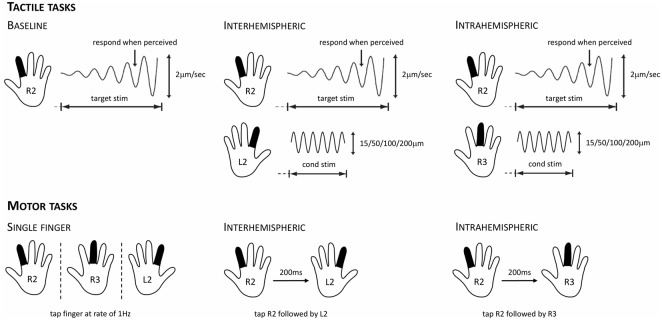
Overview of the tactile stimulation and motor protocols used for evaluating cortical inhibition. For the tactile tasks, participants were instructed to respond as soon as they felt the vibration on their right index finger (R2). This target stimulus had a starting amplitude of 0 μm and then increased at a rate of 2 μm/s. In the baseline condition (left), only the target stimulus was presented. In the interhemispheric condition (center), the target stimulus was presented while concurrently a conditioning stimulus of 15, 50, 100, or 200 μm was delivered to the left index finger (L2). In the intrahemispheric condition (right), the target stimulus was presented while concurrently a conditioning stimulus of 15, 50, 100, or 200 μm was delivered to the right middle finger (R3). For the motor tasks, participants were instructed to tap their fingers in synchrony with stimuli presented on the screen. For the R2, R3, and L2 single-finger conditions (left), they had to tap at a rate of 1 Hz. For the interhemispheric condition (center), they had to tap R2 followed by L2 after a 200 ms lag; intertap interval for a given finger remained at 1,000 ms. For the intrahemispheric condition (right), they had to tap R2 followed by R3 after a 200 ms lag.

The inhibition task consisted of two blocks of 16 trials and allowed us to evaluate the effects of concurrent inter- and intrahemispheric stimulation on tactile processing. Participants were instructed to click the mouse when they perceived a vibration on their right index finger (R2; see Figure 1). The target stimulus that was delivered to R2 had a starting amplitude of 0 μm and then increased at a rate of 2 μm/s (see dynamic threshold task), and was delivered at a frequency of 25 Hz. The delivery of this target stimulus was accompanied by the delivery of a concurrent stimulus either to the left index finger (L2; interhemispheric) or the right middle finger (R3; intrahemispheric). In line with previous work (Zhang et al., 2011; Nguyen et al., 2013), this so-called conditioning stimulus was delivered at a frequency of 25 Hz and had an amplitude of either 15, 50, 100, or 200 μm (four trials of each amplitude, randomly ordered). The inter-trial interval (ITI) was 5 s and each trial started with a variable delay period that did not involve stimulation. As in the dynamic threshold task, the stimulus amplitude at the time of the participant’s response was recorded for each trial.

#### Motor Tapping Task

Participants performed the motor tapping task while lying supine in a 3T GE MRI scanner (MRI data not included in the present report). They viewed four horizontally aligned blank squares, two to the left and two to the right of a crosshair positioned in the center of the visual display. Participants were informed that these four squares corresponded to the index and middle fingers of each hand. They were instructed to tap the corresponding finger as fast as possible once the square lit up with a (blue or orange) color, and then to synchronize their finger taps with each illumination, which occurred at a rate of 1 Hz for the duration of the 20 s-block. For example, if the rightmost square lit up, they were to tap their right middle finger.

Participants performed five different finger-tapping conditions (see Figure 1). In three conditions, participants were instructed to tap with a single finger, either R2, R3, or L2. In two other conditions, they had to tap R2 followed by their right middle finger (R2R3; intrahemispheric), or R2 followed by their left index finger (R2L2; interhemispheric). The blue and orange colors of the squares represented whether the condition was a single- or two-finger condition, respectively (counterbalanced across participants). After completing a practice run, participants performed two experimental runs of the motor task. During each run, each of the five conditions occurred twice (randomly ordered) and lasted for 20 s experimental blocks. The experimental blocks were interleaved with 12 s rest blocks during which participants were instructed to gaze at a fixation cross in the center of the visual display. The ITI in the single- and two-finger conditions was 1 s, and the lag between the successive illuminations in the two-finger conditions was 200 ms.

### Data Processing and Statistical Analyses

#### Tactile Tasks: RT and Thresholds

For the sRT and cRT tasks, we determined the mean RTs across all trials for each participant. RTs deviating more than 3SD from a participant’s mean were omitted from the analyses (1.8% of all trials). For the cRT task, we additionally excluded RTs of trials with incorrect responses (mean accuracy = 93%; accuracy in the sRT task was always 100% as there was only one response option). We also determined the static and dynamic thresholds for each participant. The static threshold was defined as the smallest constant-amplitude stimulus that a participant could detect correctly, whereas the dynamic threshold was defined as the minimum detectable amplitude of a stimulus that gradually increased in intensity starting from 0 (e.g., Zhang et al., 2011). For the dynamic threshold task, data of two participants who had fewer than five correct responses were excluded from the analyses. Linear regression analyses on participants’ performance in these tasks were run to evaluate the association of age with tactile performance. As these analyses were performed for each of the four tasks, the alpha level for the regressions analyses was set at a statistical threshold of 0.0125 to correct for multiple comparisons.

#### Tactile Tasks: Inhibition

For the tactile inhibition tasks, one participant responded correctly to only 53% of the trials and was therefore excluded from the analyses. For the remaining participants (mean accuracy = 97.5%) we averaged the stimulus amplitude at the time of their response on correct trials to obtain individual DTs. This was done as a function of concurrent stimulation amplitude (15, 50, 100, or 200 μm) and condition (intra vs. interhemispheric). We normalized the observed DTs to correct for individual baseline differences, by determining the percent change compared to baseline performance for each participant. Baseline performance was defined as the mean DT on R2 trials in the dynamic DT task, where R2 was stimulated without concurrent stimulation to another finger. To test the effect of concurrent stimulation to either R3 (intrahemispheric) or L2 (interhemispheric) on detection of a target stimulus on R2, we compared R2 baseline performance with R2 performance during presentation of a 15 μm conditioning stimulus. We ran a regression analysis on the normalized DT %change scores to evaluate the extent to which age determined the change in tactile performance. To examine the effect of conditioning stimulus amplitude on DT and whether this was age-dependent, we then performed repeated measures ANCOVAs on DT %change with amplitude (4; 15, 50, 100, 200 μm) as a within-subject variable and age as a covariate. Significant interactions were followed up by first calculating for each participant the slope of DT %change across the four amplitude conditions within the inhibition tasks, and then performing a regression analysis with age as the independent variable and slope as the dependent variable. As these analyses were performed for both the inter- and intrahemispheric condition, the alpha level was set at a statistical threshold of 0.025 to correct for multiple comparisons.

#### Motor Tasks

Performance in the motor tapping task was evaluated by calculating the mean between-finger lag and the variability of this lag for each tapping condition. The between-finger lag reflects the time between two successive taps with the same finger (averaged across all three single-finger conditions), or between the asynchronous taps with R2 and either R3 or L2 in the intra- and interhemispheric conditions, respectively. The variability of the between-finger lag reflects the SD of the lag and is indicative of participants’ ability to maintain the 1 s lag in the single-finger blocks and the 200 ms lag in the two-finger blocks. We have used these measures previously in studies examining effects of aging on unimanual and bimanual movements, and have linked them to motor inhibitory function (Bangert et al., 2010; Fling et al., 2011). Data from four participants (aged 19, 20, 24, and 26 years) were excluded due to technical issues related to the presentation/recording software. We also excluded tapping trials when participants tapped an incorrect finger or when the average between-finger lag or variability deviated more than 2.5 SD from the overall group mean. The latter procedure resulted in the exclusion of the single-finger variability for one participant (30 years) and variability for tapping with two fingers on the same hand for another (72 years). To assess whether age is predictive of motor tapping performance, we ran regression analyses with age as the independent variable and the between-finger lag or variability as the dependent variable.

#### Additional Analyses

To analyze the age of onset for the hypothesized changes in inhibitory function, we used locally weighted polynomial regession (LOESS) smoothing to describe the age trajectories (see Westlye et al., 2010; Chan et al., 2014). This approach is similar to spline smoothing and has been shown to be superior for describing age effects compared to models including a quadratic or cubic term, as well as being more robust to variations in the age range (Fjell et al., 2010). For the tactile data, we then determined the age at which the DT %change in the 15 μm condition started to decrease by calculating the LOESS estimated maxima. For the motor data, these maxima are not informative of changes as they only reflect the age at which performance deviates most from the 200 ms target (rather than when this deviation starts). Therefore, we determined the age of onset by identifying the inflection point for each trajectory. Finally, we performed correlation analyses to examine the relationship between motor tapping and tactile performance. Data processing and statistical analyses were performed using IBM SPSS software (version 23.0; IBM Corp, 2015), except for the age of onset analyses which were performed in R (version 3.5.1; R Core Team, 2018).

## Results

The data from one participant were excluded from analyses as this individual scored below the cut-off of 23 on the MoCA (see Carson et al., 2018); this participant did not perform the motor tasks. As such, results of the tactile tasks are reported for the 55 remaining participants and results of the motor tapping task for 33 participants (as outlined in “Motor Tasks” section). Participant demographic characteristics and performance on the cognitive and motor neuropsychological tests are listed in Table 1. Correlation analyses showed that information processing speed as reflected in DSST scores and manual motor performance as reflected in Purdue pegboard scores declined with increasing age, while age was not significantly associated with general cognitive abilities as evaluated by the MoCA.

**Table 1:** Demographic characteristics and performance on the cognitive and motor neuropsychological tests.

	Mean ± SD	Range	Correlation with age	B
Age	44.6 ± 18.4	18–76		
Gender	33 F/22 M	-		
MoCA	27.8 ± 1.6	24–30	*p* = 0.118	
Pegboard				
Right hand	14.8 ± 2.4	10.7–19.0	*r*_(55)_ = −0.67, *p* <0.001	−0.09
Left hand	13.7 ± 2.3	9–18	*r*_(55)_ = −0.60, *p* <0.001	−0.07
Both hands	11.6 ± 1.9	7.3–15.3	*r*_(55)_ = −0.55, *p* <0.001	−0.59
Assembly	34.1 ± 8.2	17.7–51.0	*r*_(55)_ = −0.72, *p* <0.001	−0.32
Digit symbol				
Version 1	59.1 ± 13.9	26–91	*r*_(54)_ = −0.78, *p* <0.001	−0.59
Version 2	60.0 ± 12.7	28–93	*r*_(54)_ = −0.69, *p* <0.001	−0.47

### Tactile Performance

**Figure 2:**
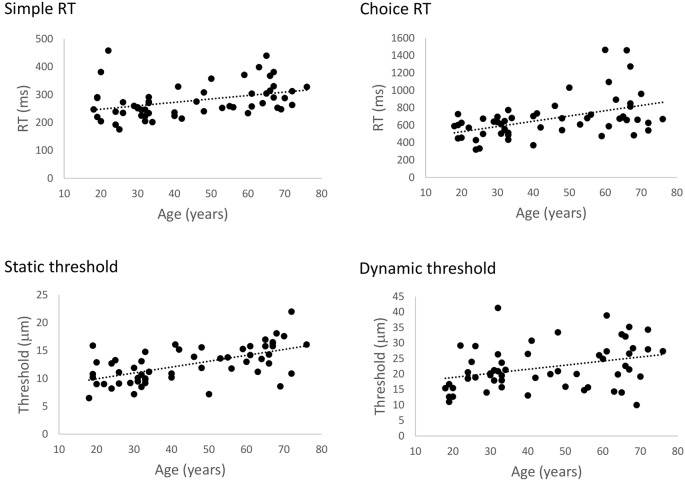
The effect of age on general tactile processing. Scatterplots illustrating the relationship between age and simple reaction time (sRT), choice reaction time (cRT), static threshold, and dynamic threshold, respectively. Performance on all four tests significantly declined with age (*p*s < 0.006).

Figure 2 illustrates the relationship between age and performance on the RT and threshold tasks. The results of our regression analyses showed that both simple and choice RT significantly increased as a function of age, Bs > 1.22, *t*s > 2.876, *p*s < 0.006. Similarly, both static and dynamic DTs were found to increase with age, Bs > 0.106, *t*s > 2.84, *p*s < 0.006. Accuracy in the cRT task did not vary as a function of age (*p* = 0.18). These findings correspond with previous observations of age-related declines in basic measures of tactile function as measured by these protocols (Zhang et al., 2011), and confirm that general tactile processing in the present participants is representative of other age samples.

**Figure 3:**
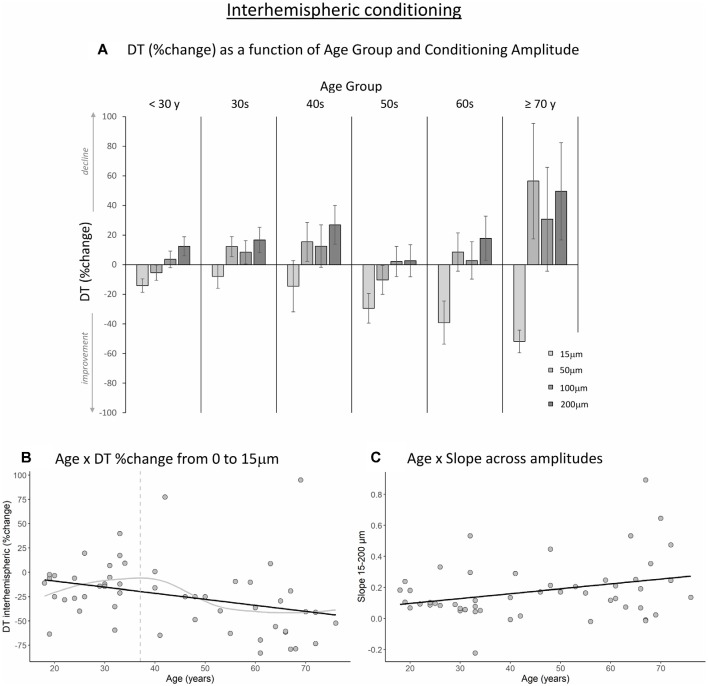
The effect of age on interhemispheric conditioning. **(A)** Detection threshold (DT; measured in %change compared to baseline) as a function of age group and amplitude of the conditioning stimulus. Data are plotted by age group for visualization purposes only; all analyses were conducted using age as a continuous variable. **(B)** The black line shows that with increasing age, DTs improved more when a conditioning stimulus (15 μm) was delivered to a contralateral homologous finger (*p* = 0.010). The gray line shows the LOESS smoothed age trajectory; the dashed line indicates the age at which changes started. **(C)** Relationship between age and the slope of DT change across the four amplitude conditions. With increasing age, DTs declined faster as a function of increasing conditioning stimulus amplitude (*p* = 0.029).

**Figure 4:**
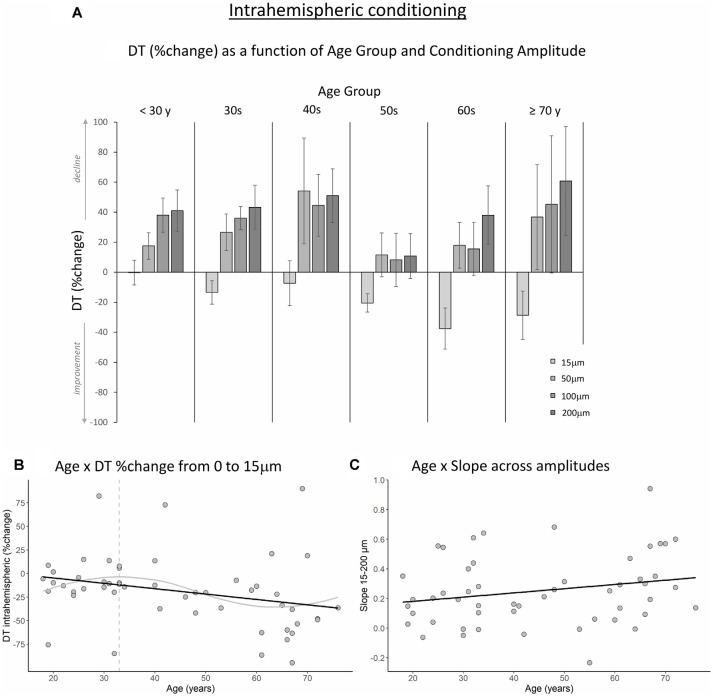
The effect of age on intrahemispheric conditioning. **(A)** DT (measured in %change compared to baseline) as a function of age group and amplitude of the conditioning stimulus. Data are plotted by age group for visualization purposes only; all analyses were conducted using age as a continuous variable. **(B)** The black line shows that with increasing age, DTs improved more when a conditioning stimulus (15 μm) was delivered to a neighboring finger (*p* = 0.016). The gray line shows the LOESS smoothed age trajectory; the dashed line indicates the age at which changes started. **(C)** The effect of increasing the conditioning stimulus amplitude on DT did not change significantly with age (*p* = 0.11).

Figures 3A, 4A show the percent change in DT compared to baseline as a function of conditioning stimulus amplitude in the interhemispheric and intrahemispheric conditions, respectively. Negative values for the 15 μm amplitudes in these figures reveal that DTs decreased with a conditioning stimulus on a neighboring finger or the contralateral homologous finger, indicative of a benefit from age declines in surround and interhemispheric inhibition. The pattern of positive values for the other amplitudes suggests that this “spillover” of neural activity across neighboring or contralateral representations resulted in increasing detection difficulties for older individuals at higher conditioning amplitudes. It should be noted that we categorized participants into separate age groups for illustration purposes in the figures only. To examine whether age was associated with the magnitude of change in DT under a 15 μm concurrent stimulation condition compared to baseline, we ran regression analyses with age as an independent variable and DT (operationalized as the percent change compared to baseline DT) as the dependent variable. The results showed that age significantly predicted the DT change in both the interhemispheric condition, B = −0.071, *t* = −2.68, *p* = 0.010, and the intrahemispheric condition, B = −0.68, *t* = −2.507, *p* = 0.016. More specifically, as Figures 3B, 4B illustrate, the negative slopes show that higher age is associated with greater improvements (decreases) in DT. The results of the LOESS analyses showed that declines in inhibition started at 37 years for the interhemispheric condition and at 33 years for the intrahemispheric condition.

Next, to examine how the amplitude of the conditioning stimulus affected DT and whether this impact differed due to age, we performed repeated-measures ANCOVAs on DT %change compared to baseline R2 with amplitude (4; 15, 50, 100, 200 μm) as a within-subject variable and age as a covariate. The results showed a significant interaction between amplitude and age for the interhemispheric condition, *F*_(3,150)_ = 10.40, *p* < 0.001. We observed a similar trend for the intrahemispheric condition, *F*_(3,150)_ = 3.02, *p* = 0.044, although this effect did not reach our corrected statistical threshold. There were no main effects of amplitude or age (*p*s > 0.19). To further investigate the interactions, we calculated the slope of DT %change across amplitude conditions 15–200 μm for each participant. This was done separately for the inter- and intrahemispheric conditions. We then performed regression analyses with age as the independent variable and slope as the dependent variable. The results showed that the slope was associated with age for the interhemispheric condition, B = 0.003, *t* = 2.24, *p* = 0.029. Specifically, steepness of the slope increased with age (see Figure 3C), indicating that DTs declined faster as a function of conditioning stimulus amplitude with increasing age. While the same pattern was observed for the intrahemispheric condition (see Figure 4C), results of the regression analysis showed that this association was not significant (B = 0.003, *t* = 1.61, *p* = 0.11).

### Motor Tapping Performance

**Figure 5:**
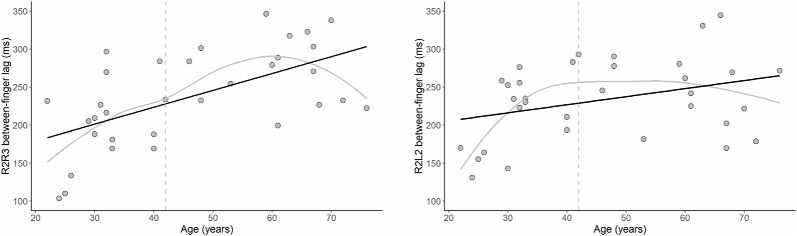
The effect of age on motor performance. Relationship between age and the between-finger lag in the motor tapping task for the intrahemispheric (left) and interhemispheric (right) conditions. Note that the optimal target lag is 200 ms. Black lines show that the duration of the lag significantly increased with age for the intrahemispheric condition (*p* < 0.001) and, while not statistically significant, a similar pattern was observed for the interhemispheric condition (*p* = 0.054). Gray lines show the LOESS smoothed age trajectories; dashed lines indicate the age at which changes started.

The results of our regression analyses revealed that the intrahemispheric between-finger lag was significantly predicted by age, B = 2.22, *t* = 4.14, *p* < 0.001. Specifically, increasing age was associated with an increased duration of the between-finger lag (see Figure 5). In addition, Figure 5 shows that for younger participants the between-finger lag was shorter than the instructed 200 ms, but gradually increased with age. This indicates that younger participants underestimated the paced inter-stimulus interval, whereas older participants overestimated the interval. The results showed a similar pattern for the relationship between age and interhemispheric between-finger lag, although this effect was not significant, B = 1.06, *t* = 2.01, *p* = 0.054. The LOESS analyses revealed that the onset of age differences was at 42 years for both intrahemispheric and interhemispheric performance. Finally, we observed no significant relationship between age and our single-finger measures (*p*s > 0.42) or intra- and inter-hemispheric SDs (*p*s > 0.34).

### Associations Between Tactile and Motor Performance

Contrary to our hypothesis, we did not observe a significant correlation between performance of asynchronous movements and tactile inhibition for either intrahemispheric (*p*s > 0.12) or interhemispheric conditions (*p*s > 0.85). To determine whether the lack of significant correlations should be interpreted as evidence for the independence of age effects in the tactile and motor systems, we reanalyzed our data using a Bayesian approach. Specifically, we performed Bayesian correlation analyses with default prior settings using JASP software (version 0.8.1; JASP Team, 2017). The Bayes factors (BF_10_) indicated that the current data provide anecdotal to moderate evidence for independence in the intrahemispheric condition (BF_10_ = 0.663 for between-finger lag duration and BF_10_ = 0.251 for variability) and moderate evidence for independence in the interhemispheric condition (BF_10_ = 0.227 for duration and BF_10_ = 0.225 for variability).

Finally, we examined whether general tactile performance was associated with motor tapping performance. The results revealed that performance of asynchronous movements of two digits on the same hand (i.e., intrahemispheric R2R3 performance) was significantly correlated with general tactile performance measures. We observed that the duration of the between-finger lag correlated with simple RT, *r*_(33)_ = 0.506, *p* = 0.003, with longer lags being associated with slower simple RTs. Similarly, a longer duration of the between-finger lag was associated with slower cRTs, *r*_(33)_ = 0.413, *p* = 0.0017. There were no significant associations between our general tactile measures and either single-finger (*p*s > 0.10) or interhemispheric motor measures (*p*s > 0.18).

## Discussion

Here, we provide new evidence regarding the effects of healthy aging on surround and contralateral inhibition in the somatosensory and motor systems. A group of participants spanning an age range from 18 to 76 years performed a battery of tactile and motor tasks, which allowed us to evaluate differences in sensorimotor performance across the lifespan. Consistent with prior studies we observed that basic tactile function declined as a function of age, thus confirming that general tactile processing in our sample is representative of that observed in similar age samples (e.g., Lin et al., 2005; Zhang et al., 2011). Moreover, our results reveal novel evidence for age differences in tactile inhibitory function. We observed that older age was associated with greater improvements (decreases) in DT when a low-amplitude stimulus was simultaneously applied to the neighboring or contralateral finger. This suggests that in older adults the receptive field of the target finger was excited by stimulation on a neighboring finger such that detection of the stimulus was facilitated, whereas in younger adults the receptive field was inhibited by concurrent stimulation and consequently this hindered stimulus detection. These findings are indicative of age-related declines in both interhemispheric and intrahemispheric inhibition in the tactile system.

We found similar support for age-related declines in motor inhibition, with effects more pronounced for the intrahemispheric condition than the interhemispheric condition. Specifically, we observed that older age was associated with an increased duration of the between-finger lag during unimanual tapping. The results further showed that simple and choice RT were associated with intrahemispheric inhibition in the motor tapping task. RT measures are thought to reflect processing speed, an ability that is known to decline with age (Salthouse, 1996). Finally, in contrast to our hypothesis, the results showed that our tactile and motor inhibition measures were not significantly correlated with each other across participants, suggesting at least some degree of independent aging effects.

Notable strengths of the present study are that we assessed both tactile and motor processing and included participants across a wide range of ages, whereas previous studies have typically only examined one domain and compared extreme age groups (e.g., Zhang et al., 2011; Fujiyama et al., 2016; Mooney et al., 2017). The current design allowed us to examine not only age differences but also to evaluate when in the lifespan these differences begin to emerge. We found that declines in inhibitory function emerged around the 40s, an age of onset comparable to that described for other domains. For example, Boisgontier et al. (2018) reported that impairments in bimanual coordination start in the 40s. Similarly, a prospective cohort study examining cognitive declines in relationship to aging concluded that changes in this domain also start in the 40s (Singh-Manoux et al., 2012). However, there are also indications that cognitive declines may not be evident until later in life, with longitudinal evidence suggesting that most changes start in the 60s (Nyberg et al., 2012; but see Park et al., 2002 for cross-sectional comparisons).

Our data provide evidence that aging affects tactile and motor surround and interhemispheric inhibition, processes known to be mediated by GABA neurotransmission (Tremere et al., 2001; Beck and Hallett, 2011). Previous work using magnetic resonance spectroscopy (MRS) in human subjects reported a significant, negative, linear association of GABA concentration in parietal and prefrontal cortical regions with age in participants ranging in age from 20 to 80 years old (Gao et al., 2013). Accumulating evidence also points to inhibitory GABA neurotransmission as a potential factor in age-related declines in both somatosensory and motor performance. Specifically, studies using MRS have demonstrated that individual differences in GABA concentration in the sensorimotor cortex and supplementary motor area, respectively, are associated with tactile (Puts et al., 2011, 2014) and motor (Boy et al., 2010) function in young adults. In addition, individual differences in regional GABA concentration are associated with cognitive function in older adults (Porges et al., 2017; Simmonite et al., 2019). Our study extends these findings by showing that age-related decreases in tactile surround and interhemispheric inhibition actually *benefit* performance for older adults when low-amplitude stimulation is applied to a neighboring or contralateral finger. One interpretation of this finding is that age-related reductions in GABA-mediated inhibition reflect an underlying compensatory mechanism aimed at enhancing neural plasticity to adapt to the multifaceted declines of increasing age (Caspary et al., 1999; Grachev et al., 2001; Hoekzema et al., 2012). Such a perspective is based on the idea of homeostatic disinhibition, which compensates for an overall reduced excitatory drive by recruiting additional cognitive reserve during aging (Gleichmann et al., 2011). While our data thus support the notion that age-related changes in tactile processing may be mediated by GABA concentration, this relationship is less straightforward for motor function. We observed that motor tapping performance was correlated with general tactile measures assumed to be indicative of processing speed (i.e., sRT/cRT). Therefore, age-related declines in motor control could be associated with underlying reductions in both processing speed and GABA-mediated inhibitory function.

When considered in the context of the associated physiology, the present behavioral results suggest that declines in GABA neurotransmission may underlie age differences in inhibition in both the motor and sensory system. While we propose a single mechanism for age effects in motor and sensory processing, it should be noted that our results showed that measures of inhibition in these systems were not significantly correlated across participants. This may indicate that individual differences in declines of GABA concentration are not uniform across the brain (see Greenhouse et al., 2016). An additional caveat to consider concerning GABA concentration as a mechanism underlying age-related performance effects is that the documented relationship between age and GABA has been mixed to date. Specifically, whereas some studies observed age-related declines in GABA concentration (e.g., Gao et al., 2013; Porges et al., 2017; Hermans et al., 2018a; Cassady et al., 2019), others report no significant difference between young and older adults (e.g., Maes et al., 2017; Mooney et al., 2017; Hermans et al., 2018b). In addition, the present finding that higher conditioning amplitudes resulted in increased detection difficulty for older individuals suggests a fine line between compensation (or, paradoxical benefits) and loss of function. Future studies should systematically examine whether changes in GABA concentration indeed mediate age-related changes in tactile and motor inhibitory function, and investigate the neural mechanisms underlying the point at which compensation transitions to loss of function. It may be that task demands or context determine whether an age-effect is beneficial (i.e., compensatory) or impairing (see Reuter-Lorenz and Cappell, 2008).

Some limitations of this work should be acknowledged. First, the cross-sectional design does not allow for causal inferences about the relationship between aging and alterations in inhibitory function. Similarly, our correlational results clearly demonstrate associations, but they cannot conclusively establish directional effects. Future studies should, therefore, use longitudinal designs to measure age-related changes in inhibitory function in different domains. In addition, middle ages were relatively underrepresented in our sample as the recruitment of participants from this working population is difficult. Nevertheless, the scatter plots indicate that the data from the middle-aged adults in our sample are orderly in that they lie between the younger and older subjects, both for the inhibition measures and measures of basis tactile function. This suggests that while small, our middle-aged sample is representative. Another limitation is that we did not directly measure GABA concentrations with MRS; consequently, our suggestions about neurochemical mechanisms underlying the observed age-related effects on tactile and motor inhibitory function are speculative. However, our study utilized an inhibition measure that has been clearly linked to GABA (Tremere et al., 2001). Future research should further examine the neural mechanisms underlying age-related effects in the somatosensory and motor systems, by investigating whether brain activation patterns during task performance in these domains are increasingly overlapping with older age. In addition, studies should assess potential associations between GABA concentration and performance related to inhibitory function in the tactile and motor systems.

In conclusion, our findings demonstrate that aging impacts tactile and motor inhibitory function. However, the age effects we document in the somatosensory and motor system were found to be relatively independent, suggesting that changes in inhibitory function may not occur uniformly across the brain. We propose that age-related declines in inhibition may be related to changes in GABA neurotransmission, although future studies should further address this by relating behavioral indices of inhibition to GABA metrics.

## Data Availability

The datasets generated for this study are available on request to the corresponding author.

## Ethics Statement

The written informed consent was obtained from all participants, who received monetary compensation for their participation. The study was approved by the Institutional Review Board of the University of Michigan.

## Author Contributions

PR-L, MT, and RS conceived and designed the study. KC organized and performed data collection, and contributed to the study design and data interpretations. MR contributed to the study design and analyzed the data. MR and RS wrote the first draft. All authors reviewed the manuscript and approved the final version.

## Conflict of Interest Statement

MT is the co-founder of Cortical Metrics, a company licensed by the University of North Carolina to distribute devices that supported the methods reported in this article. The remaining authors declare that the research was conducted in the absence of any commercial or financial relationships that could be construed as a potential conflict of interest.

## Footnotes

^1^http://www.vislab.ucl.ac.uk/cogent.php
